# Treatment burden, adaptation, and support needs during exclusive enteral nutrition in children with Crohn’s disease: a qualitative meta-synthesis

**DOI:** 10.3389/fnut.2026.1920373

**Published:** 2026-09-16

**Authors:** Fangyuan Wang, Xianhong Li, Yinghui Guan, Jiaqi Tian, Bing Huang, Jianhui Xie

**Affiliations:** 1 Xiangya School of Nursing, Central South University, Changsha, China; 2 Department of Orthopedics, The Affiliated Children’s Hospital of Xiangya School of Medicine, Central South University (Hunan Children’s Hospital), Changsha, Hunan, China; 3 Hospital Administration Office, The Affiliated Children’s Hospital of Xiangya School of Medicine, Central South University (Hunan Children’s Hospital), Changsha, Hunan, China

**Keywords:** adaptation, children and adolescents, Crohn’s disease, exclusive enteral nutrition, multi-perspective synthesis, qualitative meta-synthesis, support needs, treatment burden

## Abstract

**Objective:**

To synthesise child and adolescent accounts and caregiver and healthcare-professional perspectives on treatment burden, adaptation, and support needs during exclusive enteral nutrition (EEN) and early food reintroduction in paediatric Crohn’s disease.

**Design:**

A multi-perspective qualitative meta-synthesis using the Joanna Briggs Institute (JBI) meta-aggregative approach.

**Data sources:**

PubMed, Embase, Web of Science Core Collection, the Cochrane Library, Scopus, China National Knowledge Infrastructure, Wanfang, VIP, and the Chinese Biomedical Literature Database were searched from inception to 30 January 2026. Reference lists were examined for eligible reports and theses. A structured update of PubMed and targeted publisher, index, citation, and Chinese-language title sources was conducted on 20 July 2026. In response to peer review, CINAHL with Full Text (EBSCOhost) was searched from inception to 10 August 2026.

**Review methods:**

Qualitative studies and mixed-methods reports with an identifiable qualitative component were eligible. Direct child or adolescent accounts were distinguished from caregiver proxy accounts and healthcare-professional perspectives, with findings labelled by treatment phase. Two reviewers independently screened reports, extracted findings and illustrations, and appraised qualitative components using the JBI checklist. Findings were assigned JBI credibility levels, synthesised inductively, assessed using ConQual, and examined in two sensitivity analyses: one restricted to four child-focused EEN reports and one excluding the two survey-based reports.

**Results:**

Six studies were included after reassessment led to the exclusion of one broader paediatric inflammatory bowel disease dietary study and one parent survey without an identifiable qualitative analysis. All six contained direct child or adolescent data; two also included caregiver data and one included healthcare-professional data. Twenty-two findings were grouped into eight categories and three synthesised findings: multiple burdens of EEN, reported adaptation and changing perceptions during EEN, and support needs across EEN and early food reintroduction. Both sensitivity analyses retained all three synthesised findings and all eight categories. ConQual confidence was moderate for each.

**Conclusion:**

The evidence suggests that children and adolescents may experience EEN as demanding and socially visible. Caregiver and professional perspectives add context but should not replace children’s accounts. Practice implications concerning early assessment, family support, school coordination, and food-reintroduction guidance are proposed considerations rather than established interventions.

## 1 Introduction

Crohn’s disease is a chronic, relapsing inflammatory bowel disease that may occur during childhood or adolescence and affect multiple aspects of long-term health. Children with Crohn’s disease frequently experience gastrointestinal symptoms, including abdominal pain and diarrhoea, as well as extra-intestinal consequences such as malnutrition, impaired linear growth, delayed puberty, fatigue and reduced quality of life (1, 2). Unlike adult-onset disease, paediatric Crohn’s disease occurs during a critical developmental period in which disease activity, nutritional compromise and treatment demands may disrupt growth, schooling, family routines and psychosocial development. Therefore, disease management in children requires attention not only to inflammatory control but also to nutritional recovery, developmental needs and everyday treatment burden.

Exclusive enteral nutrition (EEN) is recommended in international paediatric Crohn’s disease guidelines as a first-line induction therapy for selected children with active luminal disease (2). It can promote mucosal healing, improve nutritional status and support growth while avoiding corticosteroid-related adverse effects (1, 2). Real-world data also suggest that exclusive enteral nutrition remains an important induction strategy in paediatric Crohn’s disease care, including in evolving treatment pathways where nutritional therapy may be combined with biologics or other maintenance strategies (3). However, the clinical effectiveness of EEN depends heavily on sustained adherence, which requires children to replace ordinary food with a nutritionally complete formula for several weeks and to maintain this regimen across home, school and social settings.

This means that, unlike many pharmacological therapies, EEN is experienced not only as a medical treatment but also as a highly visible and restrictive daily Practice. Children may have to repeatedly consume formula with limited flavour options, tolerate smell, texture and gastrointestinal discomfort, resist food cravings and temporarily withdraw from ordinary eating Practice (4, 5). The interruption of normal eating may be particularly challenging because food is closely connected with family interaction, peer belonging, school life and social identity. For children and adolescents, drinking formula, avoiding shared meals or carrying treatment-related supplies may expose their illness to others and increase embarrassment, perceived difference and social pressure.

As the care pathway for EEN indicates, successful treatment requires more than prescribing formula; it also depends on careful preparation, monitoring, food reintroduction planning and continuing nutritional support (6). In Practice, however, children and families may receive insufficient information about how to implement the regimen, manage symptoms, respond to difficulties and transition back to ordinary food. Parents or caregivers often become the main coordinators of treatment, responsible for supervision, encouragement, dietary arrangements and communication with healthcare professionals (7, 8). At the same time, excessive control may conflict with adolescents’ need for autonomy, and inadequate professional follow-up may increase uncertainty and treatment burden. School-related barriers, including limited time, lack of private space for formula preparation and fear of peer attention, may further compromise adherence and continuity of care (9).

Understanding EEN requires attention to several levels of experience. Children and adolescents can report the bodily, emotional, and social consequences of treatment directly; caregivers can describe family management and implementation work; and healthcare professionals can identify service-level barriers and guidance needs. These perspectives are complementary but not interchangeable. A multi-perspective synthesis should therefore retain the source of each finding and avoid presenting proxy or professional interpretations as if they were direct child accounts.

Previous reviews of paediatric Crohn’s disease have mainly focused on clinical effectiveness, dietary strategies, treatment protocols, or EEN care pathways. Individual qualitative and mixed-methods reports have described formula tolerance, food restriction, school participation, family management, and early food reintroduction, but the evidence is dispersed across participant groups and treatment phases. A transparent synthesis is needed to distinguish children’s direct accounts from caregiver and professional perspectives while identifying areas of convergence and difference.

Therefore, this multi-perspective qualitative meta-synthesis aimed to analyse and aggregate evidence on treatment burden, adaptation, and support needs during EEN and early food reintroduction in children and adolescents with Crohn’s disease. The review addressed four questions: (1) What burdens are reported directly by children and adolescents during EEN? (2) How do children and adolescents describe adaptation over the treatment course? (3) What complementary implementation and support issues are reported by caregivers and healthcare professionals? and (4) How do findings vary by perspective and treatment phase?

## 2 Materials and methods

### 2.1 Study design

A multi-perspective qualitative meta-synthesis was conducted to integrate EEN-related evidence from primary qualitative studies and mixed-methods reports with extractable qualitative data. PRISMA 2020 guided reporting of the systematic review process, and the Enhancing Transparency in Reporting the Synthesis of Qualitative Research (ENTREQ) statement was used to strengthen reporting of qualitative synthesis methods (10, 11). The completed ENTREQ reporting checklist is provided in Supplementary Table S5.

The JBI meta-aggregative approach was selected because the purpose was to remain close to the findings reported in the primary studies and produce transparent, practice-oriented statements rather than develop a new explanatory theory (12, 13). Findings were extracted with their illustrations, assigned credibility levels, grouped by similarity of meaning, and aggregated into categories and synthesised findings. Consequently, the results should be interpreted as an organised aggregation of reported experiences across perspectives and treatment phases, not as causal conclusions or a new theoretical model.

The review was not formally registered, and no separate review protocol was publicly published. Before full-text screening and data extraction, the review team developed an *a priori* methodological plan covering the review questions, eligibility criteria, search strategy, appraisal, extraction, and JBI meta-aggregation. The eligibility criteria and synthesis method were finalised before full-text screening. During screening, one operational clarification was documented: a full-text thesis could be included when it met all substantive eligibility criteria because publication type was not an exclusion criterion. The Spiers (14) thesis was identified through reference-list searching, verified in the Theses Canada catalogue, obtained in full text, and appraised using the same criteria as journal reports. During peer-review revision, eligibility was reassessed against the prespecified phenomenon and qualitative-data requirements: Chuong et al. (15) was removed because EEN-specific findings could not be isolated, and Mehta et al. (7) was removed from meta-aggregation because the report did not describe an identifiable qualitative analytic component. These eligibility reassessments did not change the review questions or planned synthesis method. Source-specific, ConQual, and sensitivity analyses were added to improve transparency.

### 2.2 Search strategy

Nine electronic databases were searched from inception to 30 January 2026: PubMed (NCBI), Embase (Elsevier), Web of Science Core Collection (Clarivate), the Cochrane Library (Wiley), Scopus (Elsevier), China National Knowledge Infrastructure, Wanfang, VIP, and the Chinese Biomedical Literature Database (SinoMed). The database-specific strategy, platform, search date, controlled vocabulary and free-text terms, field codes, Boolean operators, limits, and number retrieved are provided in Supplementary Table S1. Reference lists of included reports and relevant reviews were examined to identify additional full-text reports and grey literature. The 2005 master’s thesis was identified through reference-list searching, verified in the Theses Canada catalogue, and obtained as a complete 131-page full text. A structured supplementary update was conducted on 20 July 2026 in PubMed and through targeted publisher websites, index/citation searching, and Chinese-language title searching for records published or indexed after 30 January 2026; the source categories, dates, procedures, and search strings used in this update are reported in Supplementary Table S1. In response to peer review, CINAHL with Full Text (EBSCOhost) was additionally searched from inception to 10 August 2026 using a Boolean strategy adapted from the original PICoS concepts; 154 records were retrieved before deduplication. APA PsycINFO was considered but was not available through the authors’ institutional database access. The institutional portal provided APA PsycArticles and the Psychology and Behavioral Sciences Collection, but these were not treated as equivalent substitutes for PsycINFO.

The search strategy was developed using the PICoS framework. Population terms covered children, adolescents, paediatric patients, caregivers, and healthcare professionals when their data directly concerned a child’s EEN pathway. Phenomenon terms included EEN, liquid diet, formula diet, polymeric formula, elemental formula, oral feeding, nasogastric feeding, tube feeding, treatment experience, adaptation, acceptability, feasibility, persistence, completion, and support needs. The context included inpatient, outpatient, home, school, follow-up, treatment completion, and early food reintroduction. Study-design terms covered qualitative research, interviews, focus groups, phenomenology, thematic analysis, content analysis, and mixed methods.

Controlled vocabulary and free-text terms were combined with Boolean operators and adapted to each database. Eligibility was restricted to English- or Chinese-language full-text reports, but no publication-date or publication-type limits were applied at the search stage. A formal PRESS peer review of the search strategy was not undertaken (16). The 20 July 2026 supplementary update used PubMed together with targeted publisher, index/citation, and Chinese-language title searches rather than a complete rerun of every licensed database. CINAHL was subsequently added during peer-review revision. Because APA PsycINFO was not available through institutional access and the July update did not constitute a full rerun of all licensed databases, some relevant psychology-indexed or newly indexed records may have been missed.

### 2.3 Eligibility criteria

Reports were included when: (a) participants were children or adolescents with Crohn’s disease undergoing or having completed EEN, or caregivers/healthcare professionals providing data directly related to the child’s EEN pathway; (b) the report addressed experiences, perceptions, adaptation, acceptability, feasibility, persistence, completion, support needs, or the early transition to food reintroduction; (c) qualitative data were collected through interviews, focus groups, open-ended survey questions, or another identifiable qualitative method; (d) an identifiable qualitative analytic process and extractable qualitative findings were reported separately from quantitative results; and (e) a full-text peer-reviewed report or thesis was available in English or Chinese. Direct child/adolescent findings, caregiver proxy findings, and professional/service-level findings were extracted and labelled separately.

Open-ended questionnaire reports were eligible only when the free-text component had an identifiable qualitative analytic process and yielded extractable author-level findings linked to respondent text. The unit of meta-aggregation was the finding-illustration pair rather than the data-collection format. Survey-based free-text data were therefore not treated as equivalent in depth to in-depth interviews; they contributed only where the reported qualitative finding could be traced directly to an open-ended response or other supporting illustration, and their more limited contextual depth was considered during appraisal and interpretation.

The principal treatment phases were active EEN, retrospective reflection after EEN completion, and early food reintroduction. Reports focused exclusively on general IBD dietary practices, long-term maintenance, relapse management, or post-treatment nutrition without an identifiable link to EEN were excluded. Chuong et al. (15) was excluded because participants were not undergoing a prescribed EEN intervention and EEN-specific qualitative findings could not be isolated. Mehta et al. (7) was retained as contextual background evidence but excluded from meta-aggregation because it reported a structured parent survey and chart comparison without a clearly described qualitative analytic method or finding-to-illustration pairs.

Reports were excluded if they were conference abstracts, reviews, commentaries, editorials, duplicate publications, unavailable in full text, unrelated to the EEN phenomenon, or lacked extractable qualitative findings. Methodological quality was not used as a stand-alone exclusion criterion. No full-text report was excluded solely because of its JBI appraisal results.

### 2.4 Study selection

All retrieved records were imported into EndNote for reference management and duplicate removal. Two reviewers (FW and YG) independently screened the titles and abstracts of all records according to the eligibility criteria. Potentially relevant studies were then retrieved in full text and independently assessed by the same reviewers. Reasons for exclusion at the full-text stage were recorded.

Disagreements during title and abstract screening or full-text assessment were resolved through discussion. When consensus could not be reached, JX acted as the third reviewer. The study selection process is presented in a PRISMA flow diagram.

### 2.5 Quality appraisal

Two reviewers (Fangyuan Wang [FW] and Yinghui Guan [YG]) independently appraised the qualitative component of each included report using the JBI Critical Appraisal Checklist for Qualitative Research. For mixed-methods or survey-based reports, appraisal was restricted to the qualitative component because only qualitative findings, not quantitative estimates, were synthesised. The checklist was applied to the congruity of the qualitative question, data collection, analysis, representation of participant voices, researcher positioning/reflexivity, ethics, and conclusions. An additional mixed-methods appraisal tool was not used because the review did not synthesise the quantitative components. The more limited contextual depth of brief open-ended questionnaire responses was recorded as an interpretive limitation rather than being treated as an automatic exclusion criterion.

Each JBI item was rated as yes, no, unclear, or not applicable. Disagreements were resolved by discussion and, when necessary, consultation with Jianhui Xie (JX) as the third reviewer. The previously reported A/B/C labels were removed because they were not part of the standard JBI checklist and could imply an arbitrary numerical threshold. No item was designated as an automatic critical exclusion criterion. Item-level limitations informed the ConQual dependability assessment and interpretation of each synthesised finding.

### 2.6 Translation and cross-language handling

Fangyuan Wang (FW) and Yinghui Guan (YG) are native Chinese speakers proficient in reading and writing academic English. For Chinese-language reports, both reviewers independently extracted the author-level findings and supporting participant quotations in the original Chinese. FW prepared the initial English translations, and YG independently checked each translated finding and illustration against the source text for semantic equivalence, clinical terminology, and preservation of the participants’ intended meaning. Jiaqi Tian (JT) subsequently verified the final bilingual entries, source locations, and finding-to-illustration links. Disagreements were resolved through comparison with the original report and discussion; JX was consulted when consensus could not be reached.

Formal back-translation was not undertaken. Instead, line-by-line bilingual verification and consensus review were used. Source-level findings from Chinese reports were first recorded in Chinese and translated into English before cross-study aggregation. The cross-study categories and synthesised findings were developed in English to enable integration with the English-language evidence while retaining the original Chinese text in the audit trail.

### 2.7 Data extraction and synthesis

A standardised form captured publication details; participant age and sex where reported; disease and prior EEN experience; route and formula type; planned duration and completion; setting; recruitment and sampling; timing of data collection; perspective source; qualitative collection and analysis methods; and the precise EEN-related findings extracted. For mixed-methods and broader reports, only findings supported by identifiable qualitative data and directly relevant to EEN were retained. Missing contextual information was recorded as not reported rather than inferred.

FW and YG independently extracted all eligible author-level findings and their supporting illustrations from each included report. They also independently assigned a preliminary JBI credibility rating to each finding. The two extraction sets were compared item by item. Discrepancies in wording, source attribution, supporting illustration, or credibility rating were resolved by returning to the full report and discussing the evidence. JT verified the completeness and source-location accuracy of the finding-to-illustration matrix. Any disagreement that could not be resolved by consensus was referred to JX for adjudication.

The JBI meta-aggregative process proceeded in four stages (12, 13). First, each author-level finding was entered as a separate source-specific row and paired with its participant quotation, open-ended response, or other supporting illustration and its source report. Second, each finding was assigned a credibility level using the congruence between the primary author’s analytic finding and the supporting data: unequivocal when the illustration directly and unambiguously supported the finding without requiring additional reviewer inference; credible when the illustration reasonably supported the finding but some interpretive judgement remained; and unsupported when no adequate illustration was available. Brevity of a survey response alone did not determine credibility; the rating depended on the fit between the author-level finding and the reported response. Unsupported findings were retained in the audit trail but did not contribute to category formation. Third, unequivocal and credible findings with similar meanings were grouped inductively into categories. The review questions defined the scope but were not used as a deductive coding framework. Fourth, categories were aggregated into synthesised findings. Perspective source and treatment phase were retained throughout the matrix (Supplementary Table S3).

FW and YG independently reviewed the extracted findings and proposed preliminary groupings based on similarity of meaning. They then compared their groupings and jointly agreed the category structure through consensus discussion. Xianhong Li (XL) provided methodological verification of the category boundaries and the relationship between the categories and synthesised findings. Bing Huang (BH) and JT checked the final mapping to ensure that every finding remained traceable to its source illustration and that perspective and treatment-phase labels were preserved. The final eight categories and three synthesised findings were agreed by the review team; unresolved disagreements were referred to JX.

Direct child and adolescent accounts were treated as the primary evidence for lived experience. Caregiver accounts were used as proxy or family-management evidence, and professional accounts were used for implementation and service-level issues. Convergence and divergence were examined rather than assuming equivalence. Confidence in each synthesised finding was assessed using the ConQual approach, based on dependability and credibility (17). Two sensitivity analyses were undertaken. The first restricted the synthesis to four reports primarily focused on child/adolescent EEN experience (Wei et al., Brown et al., Zhang et al., and Spiers) to test whether the three-domain structure remained stable when the comparative family survey and the post-EEN food-reintroduction study were excluded. The second excluded both survey-based reports (Brown et al. and Svolos et al.) and retained only the interview/phenomenological reports (Wei et al., Zhang et al., Zhou et al., and Spiers) to test whether the category structure depended on brief open-ended questionnaire responses.

## 3 Results

### 3.1 Record retrieval results

Figure 1 shows the study-selection process. The original nine-database search identified 862 records: PubMed (*n* = 286), Web of Science (*n* = 198), Embase (*n* = 106), the Cochrane Library (*n* = 2), Scopus (*n* = 62), China National Knowledge Infrastructure (*n* = 31), Wanfang (*n* = 90), VIP (*n* = 20), and the Chinese Biomedical Literature Database (*n* = 67). After removal of 162 duplicates, 700 unique database records remained; 681 were excluded at title/abstract screening and 19 database reports proceeded to full-text assessment.

**Figure 1:**
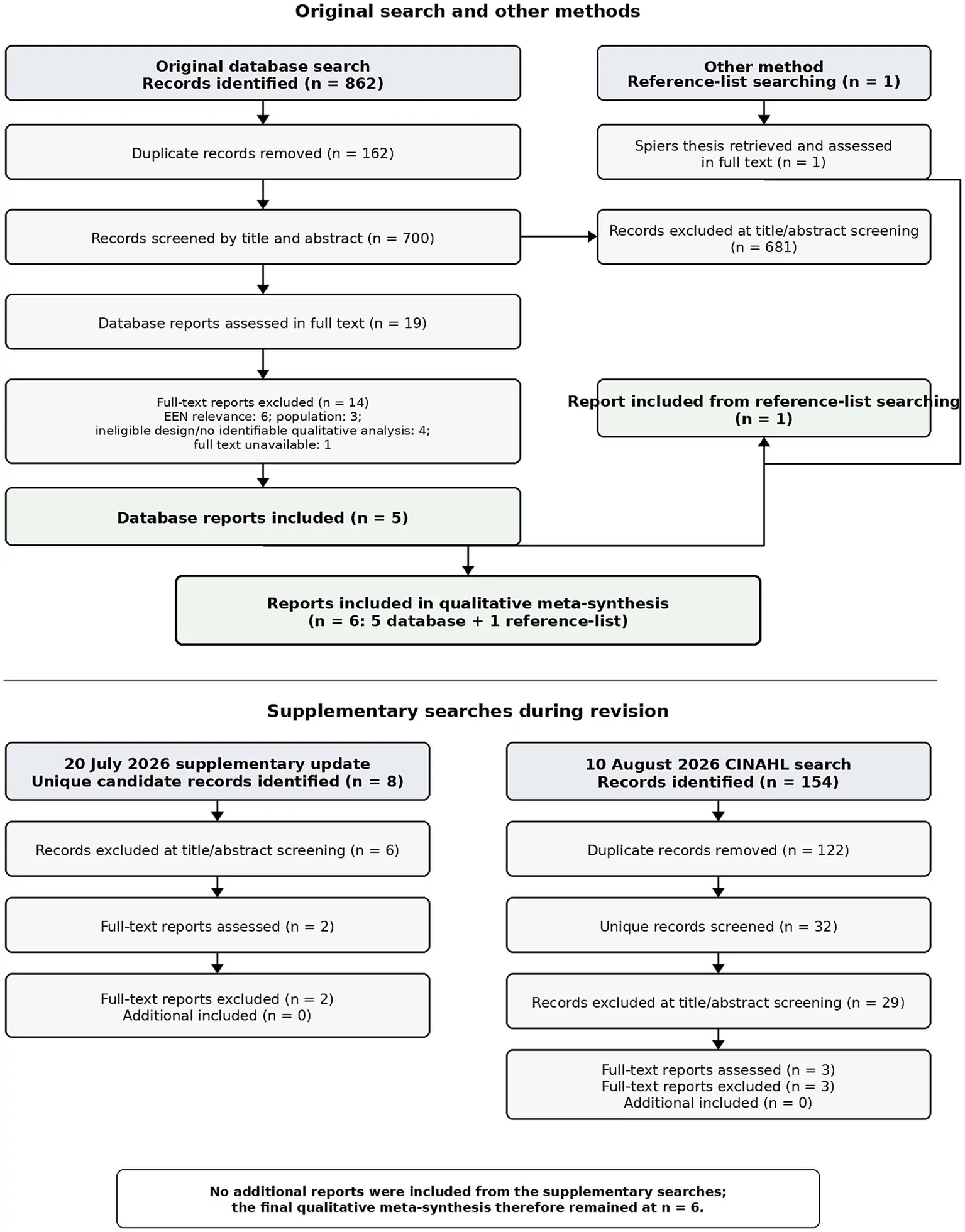
PRISMA 2020 flow diagram of study identification, screening, eligibility assessment, and inclusion.

Reference-list searching identified one additional report, the Spiers (14) thesis, which was retrieved and assessed in full text. Fourteen reports from the original search were excluded at full-text assessment: not sufficiently relevant to the EEN phenomenon (*n* = 6), target population not eligible (*n* = 3), ineligible design or no identifiable qualitative analytic component (*n* = 4), and full text unavailable (*n* = 1). Five database reports and the Spiers thesis were included. The structured supplementary update on 20 July 2026 identified eight unique candidate records; six were excluded at title/abstract screening and two reports underwent full-text assessment, with both excluded. The CINAHL search on 10 August 2026 identified 154 records; after removal of 122 duplicates against the existing search library, 32 unique records were screened, 29 were excluded at title/abstract screening, and three reports underwent full-text assessment, with all three excluded. Across the original database and supplementary-update screening pathways, 740 records underwent title/abstract screening and 716 were excluded at that stage. Together with the separately identified Spiers thesis, 25 reports were assessed in full text; 19 were excluded and six reports were included in the final qualitative meta-synthesis. No additional report was added by the July update or the CINAHL search. Details of excluded, borderline, and update-search records are provided in Supplementary Table S4.

### 3.2 Quality appraisal of the included studies

All six included reports were appraised at item level. Congruity among the research question, qualitative method, data collection, analysis, and conclusions was generally adequate. Participant voices were represented most clearly in the phenomenological and interview studies, whereas the qualitative components of survey-based reports were more limited.

Common limitations were unclear philosophical positioning, limited reporting of researcher positioning or reflexivity, and incomplete ethical reporting in some reports. These limitations reduced dependability in the ConQual assessment. No report was excluded on methodological-quality grounds. Table 1 presents item-level judgements and study-specific reasons for no or unclear ratings.

**Table 1:** Item-level JBI appraisal of the qualitative component of included reports.

Study	JBI item-level ratings (Q1–Q10)	Interpretation of no/Unclear ratings
Wei et al. (18)	U Y Y Y Y Y Y Y Y Y	Philosophical perspective not explicitly stated.
Brown et al. (4)	U Y Y Y Y N N Y Y Y	Philosophy unclear; researcher positioning and reflexivity not reported; free-text responses provided less contextual depth than interview studies.
Zhang et al. (19)	U Y Y Y Y Y U Y U Y	Philosophy unclear; reflexivity and ethical reporting incompletely described.
Zhou et al. (9)	Y Y Y Y Y N Y Y Y Y	Researcher positioning not reported.
Svolos et al. (8)	U Y Y Y Y N N Y Y Y	Open-ended survey component; philosophy, positioning and reflexivity not reported; brief comments provided limited contextual depth.
Spiers (14)	U Y Y Y Y U U Y Y Y	Philosophical positioning and researcher reflexivity were incompletely reported; ethics approval and consent procedures were reported.

Y = yes; N = no; U = unclear. Q1–Q10 follow the JBI Critical Appraisal Checklist for Qualitative Research. No numerical A/B/C grade was applied, no item was used as an automatic exclusion rule, and no report was excluded solely because of appraisal results.

### 3.3 Characteristics of the included studies

The six included reports were published between 2005 and 2025 in China, New Zealand, the United Kingdom, and Canada. All six included direct child or adolescent data; two also included caregiver data, and one included healthcare-professional data. Four reports focused primarily on active or retrospectively recalled EEN experiences, one comparative family survey examined prior EEN, and one study addressed early food reintroduction after EEN.

Three reports used phenomenological designs, one used descriptive qualitative methods, and two were survey-based reports with extractable open-ended comments. Routes of administration and contextual variables were inconsistently reported. Svolos et al. included both oral and nasogastric experiences, whereas Wei et al. focused on oral EEN. Brown et al. included children aged 5–17 years who had completed an eight-week polymeric EEN course. Because age, route, formula, completion, and interview timing were missing from several reports, route- and age-specific meta-aggregation was not possible. Expanded study context is provided in Table 2 and Supplementary Table S2.

**Table 2:** Characteristics, perspective source, and treatment context of included reports (*n* = 6).

Study	Qualitative contributors and collection	Perspective source	EEN phase/Route/Context	Sampling and timing	EEN-related findings retained
Wei et al. (18), China	12 children aged 7–15 years (7 male, 5 female); semi-structured interviews analysed using Colaizzi’s seven-step method	Direct child	First induction oral EEN; disease duration 0.25–60 months; 8 active disease and 4 remission; home and school experiences	Purposive maximum-variation sampling; 30–45 min interviews during admission or follow-up after initial EEN; formula and completion status NR	Taste and gastrointestinal discomfort; school restrictions; peer attention; parental supervision; self-regulation; symptom improvement
Brown et al. (4), New Zealand	44 children aged 5.4–16.5 years (mean 11.3); 9-item questionnaire with free-text responses analysed thematically	Direct child	After completion of an 8-week polymeric EEN course; outpatient clinic; route not reported	Convenience recruitment at routine review; retrospective timing varied	Limited flavours and disliked taste; food cravings; mental challenge; improved energy; family, school and clinical support
Zhang et al. (19), China	10 children aged 8–14 years (6 male, 4 female); semi-structured interviews analysed using Colaizzi’s seven-step method	Direct child	Active EEN pathway; 4 active disease and 6 remission; hospital, home and school context; route, formula and planned duration NR	Purposive maximum-variation sampling; 20–30 min interviews in a hospital interview room; timing relative to treatment NR; children with interrupted EEN excluded	Desire for normal eating; emotional and family support; environmental barriers; school stigma and concealment
Zhou et al. (9), China	25 participants completing 24 semi-structured interviews; purposive sampling; conventional content analysis	Teenagers, caregivers and healthcare professionals	Early food reintroduction after EEN; route/formula not reported	Interviews during transition to ordinary food	Specific guidance gaps; adolescent food acceptance; caregiver control; school constraints; timing and principles of reintroduction
Svolos et al. (8), United Kingdom	29 children and 29 parents completed separate questionnaires; 51 open-ended comments were reported and grouped by respondent type	Direct child and caregiver	Previous 8-week EEN; 55% used nasogastric support; 79% completed	All families treated in 2015; median child age 13.3 years	Perceived difficulty, isolation, route-related experience, willingness to repeat EEN, preference for solid-food alternatives
Spiers (14), Canada	10 adolescents aged 13–16 years (6 male, 4 female); phenomenological in-depth interviews, member checking, and thematic analysis	Direct adolescent	All participants had previous EEN; three were receiving EEN and seven supplemental EN at interview; NG and gastrostomy routes represented; formula NR	Purposive recruitment; home or IWK Health Centre interviews; current and recalled treatment experiences reported separately where identifiable	Only findings explicitly linked to EEN were retained: food cravings and deprivation, restriction of school participation, and treatment difficulty with anticipated benefit

The 20-year publication span was considered during interpretation. Formal subgroup analysis by publication period was not appropriate because only six reports were included, but earlier findings may reflect older formula options, clinical pathways, and school accommodations.

### 3.4 Main findings of the meta-synthesis

Twenty-two supported findings were extracted from the six reports and grouped into eight categories and three synthesised findings: (1) multiple burdens of EEN; (2) reported adaptation and changing perceptions during EEN; and (3) support needs across EEN and early food reintroduction (Figure 2). Table 3 identifies the contributing perspective sources and treatment phases, while Supplementary Table S3 provides the complete source-specific finding-to-illustration audit trail.

**Figure 2:**
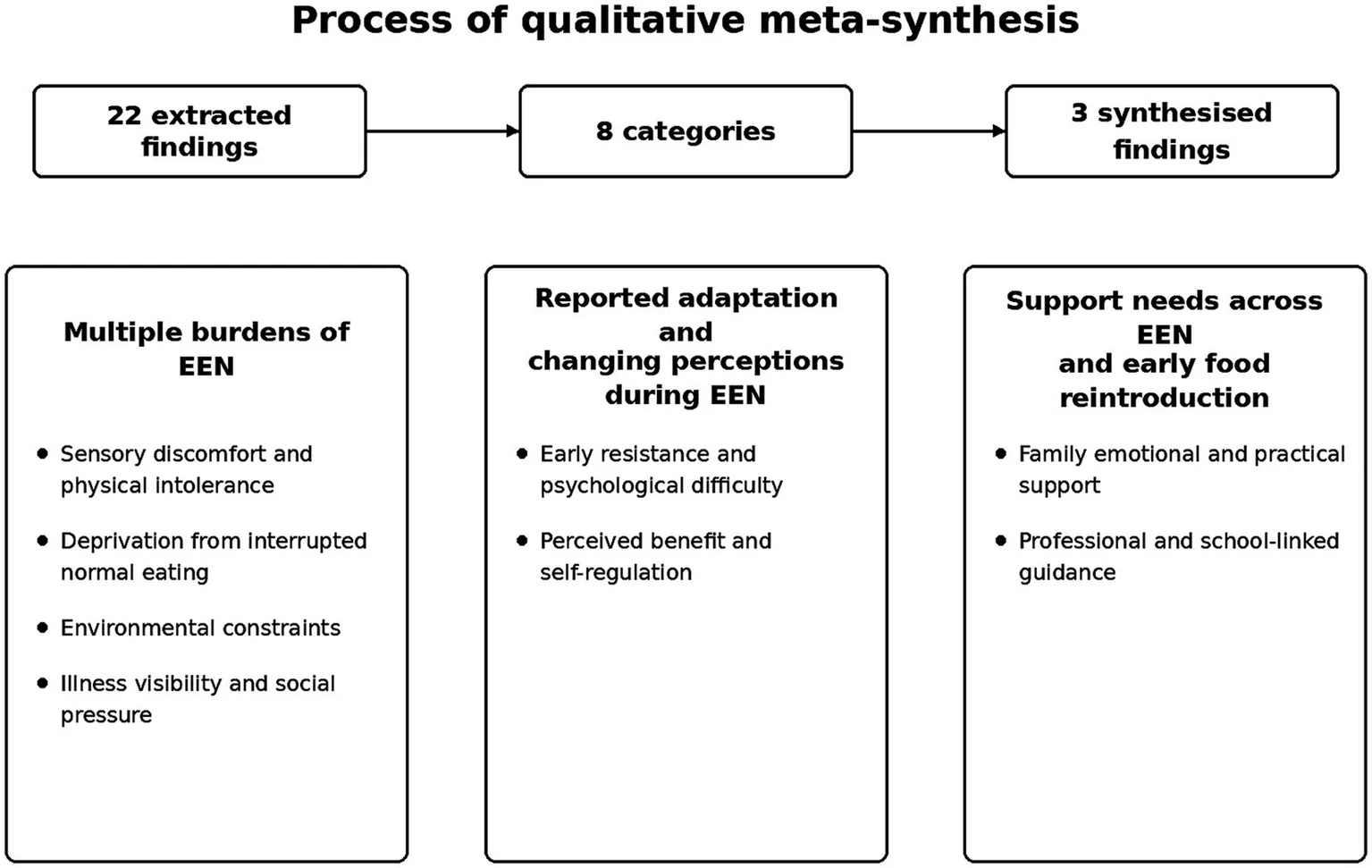
Process diagram of the synthesised findings.

**Table 3:** Categories, perspective sources, and treatment phases contributing to the synthesised findings.

Synthesised finding	Category	Representative finding	Contributing studies	Perspective source	Treatment phase
Multiple burdens of EEN	Sensory discomfort and physical intolerance	Formula fatigue, gastrointestinal discomfort, and route-related difficulty.	Wei; Svolos	Direct child plus caregiver proxy	Active or recalled EEN
Multiple burdens of EEN	Deprivation from interrupted normal eating	Cravings, loss of ordinary/family meals, and isolation.	Zhang; Svolos; Spiers	Direct child/adolescent plus caregiver proxy	Active or recalled EEN
Multiple burdens of EEN	Environmental constraints	School time, access to preparation facilities, restrictions on participation, and enteral-nutrition scheduling barriers.	Wei; Zhang; Zhou	Direct child plus caregiver context	Active EEN and early food reintroduction
Multiple burdens of EEN	Illness visibility and social pressure	Peer attention, concealment, and treatment-related social pressure.	Wei; Zhang; Spiers	Direct child/adolescent	Active EEN/tube feeding
Reported adaptation and changing perceptions during EEN	Early resistance and psychological difficulty	Mental challenge and uncertainty during treatment initiation.	Brown; Spiers	Direct child/adolescent	Active or recalled EEN
Reported adaptation and changing perceptions during EEN	Perceived benefit and self-regulation	Symptom, energy, and well-being improvement reinforced perceived treatment value and persistence.	Wei; Spiers	Direct child/adolescent	Active EEN/enteral nutrition
Support needs across EEN and early food reintroduction	Family emotional and practical support	Explanations, participation, supervision, and autonomy negotiation.	Wei; Brown; Zhou	Direct child plus caregiver proxy	Active EEN and early reintroduction
Support needs across EEN and early food reintroduction	Professional and school-linked guidance	Regular review, specific reintroduction advice, and structured professional follow-up.	Brown; Zhou	Direct child, caregiver, and professional	Active EEN and early reintroduction

### 3.5 Source-specific, sensitivity, and confidence analyses

Direct child/adolescent data supported all three synthesised findings. Caregiver data extended the family-management and feasibility dimensions, while the single professional perspective contributed to food-reintroduction and service-guidance issues. Proxy and professional findings were not presented as direct child experience. The sensitivity analysis restricted to the four child-focused EEN reports retained all three synthesised findings and all eight categories, although the breadth of food-reintroduction guidance and route-comparison evidence was reduced (Table 4). A second sensitivity analysis excluding the two survey-based reports (Brown et al. and Svolos et al.) also retained all three synthesised findings and all eight categories. Each category remained supported by at least one interview or phenomenological report, although the breadth of evidence concerning route-related experience, retrospective treatment difficulty, perceived benefit, family support, and clinical follow-up was reduced (Table 5).

**Table 4:** Sensitivity analysis restricted to direct child/adolescent EEN experience reports.

Synthesis element	Full six-report synthesis	Child-focused subset (Wei; Brown; Zhang; Spiers)	Interpretation
Three synthesised findings	All retained	All retained	Overall three-domain structure stable.
Eight categories	All retained	All retained	Category structure not dependent on caregiver/professional reports.
Food-reintroduction detail	Specific guidance, timing, caregiver control and school issues	Only general post-EEN and support comments retained	Breadth of food-reintroduction evidence depends on Zhou et al.
Service-level professional perspective	Present	Absent	Professional conclusions should be treated as contextual service evidence.

**Table 5:** Sensitivity analysis excluding the two survey-based reports.

Synthesis element	Interview/Phenomenological subset (Wei; Zhang; Zhou; Spiers)	Interpretation
Three synthesised findings	All retained	Overall three-domain structure did not depend on the two survey-based reports.
Eight categories	All retained	Every category remained supported by at least one interview or phenomenological report.
Multiple burdens of EEN	All four burden categories retained	Breadth of route-related and isolation evidence was reduced after removal of Svolos et al.
Reported adaptation and changing perceptions during EEN	Both adaptation categories retained	Spiers supported early treatment difficulty and Wei supported perceived benefit; Brown-specific retrospective free-text detail was removed.
Support needs across EEN and early food reintroduction	Both support categories retained	Wei and Zhou retained family/practical and professional guidance evidence; Brown-specific comments on family advice and clinical review were removed.

ConQual confidence was moderate for all three synthesised findings. Each finding was downgraded one level for dependability because philosophical positioning, researcher reflexivity, and related methodological reporting were incomplete in several contributing reports. No unsupported finding contributed to the synthesis. The credibility distinction was applied at the individual finding-illustration level according to the directness of fit between the primary author’s finding and its supporting quotation or open-ended response; data-collection format alone did not determine the rating. In the current audit trail, the 22 findings contributing to synthesis were judged to have direct, unambiguous supporting illustrations and were therefore retained as unequivocal, so no ConQual credibility downgrade was applied. Supplementary Table S3 provides the source report, finding, supporting illustration, source location/participant identifier where available, perspective, treatment phase, credibility rating and rationale, and the category and synthesised finding to which each item contributed (Table 6).

**Table 6:** ConQual summary of findings.

Synthesised finding	Initial ConQual level	Dependability judgement	Credibility judgement	Final ConQual rating and reasons
Multiple burdens of EEN	High	Downgraded one level	No downgrade	Moderate: downgraded one level for dependability because reflexivity and philosophical reporting were incomplete in contributing reports. No credibility downgrade: contributing findings had direct, unambiguous supporting illustrations; survey-derived items were retained only where the reported response directly supported the author-level finding. Full audit trail in Supplementary Table S3.
Reported adaptation and changing perceptions during EEN	High	Downgraded one level	No downgrade	Moderate: downgraded one level for dependability because reflexivity and philosophical reporting were incomplete in contributing reports. No credibility downgrade: contributing findings had direct, unambiguous supporting illustrations; survey-derived items were retained only where the reported response directly supported the author-level finding. Full audit trail in Supplementary Table S3.
Support needs across EEN and early food reintroduction	High	Downgraded one level	No downgrade	Moderate: downgraded one level for dependability because reflexivity and philosophical reporting were incomplete in contributing reports. No credibility downgrade: contributing findings had direct, unambiguous supporting illustrations; survey-derived items were retained only where the reported response directly supported the author-level finding. Full audit trail in Supplementary Table S3.

### 3.6 Multiple burdens of EEN

Direct accounts from children and adolescents described EEN as restrictive and demanding across bodily, emotional, practical, and social domains. Caregiver reports generally corroborated the disruption of family meals and social participation, but these proxy observations were kept distinct from children’s own statements.

Sensory discomfort and physical intolerance were supported predominantly by direct child and adolescent data. Reports described limited flavour variety, disliked taste, formula fatigue, nausea, vomiting, abdominal discomfort, and oral or tube-related difficulty. One child explained, “After drinking it a few times, I started to get tired of it; the smell made me want to vomit” [(18), participant N5]. Route-specific variation could not be synthesised consistently because administration route was incompletely reported across studies.

Interrupted normal eating generated deprivation and loss of participation. Children described food cravings, missing ordinary meals, and difficulty watching others eat. One adolescent described “dealing with the cravings of certain foods like pizza” while receiving EEN [(14), p. 64]. Family-level data reinforced this social meaning; a parent in Svolos et al. described the liquid-only diet as “very isolating at times for my child.” These sources were interpreted as complementary rather than equivalent.

Environmental constraints were most visible in school settings. Child and adolescent accounts described limited time, lack of suitable preparation facilities, and restrictions on participation. One child reported that the classroom was on the fourth floor while hot water was available only on the first floor, which “wastes time” [(19), participant N7]. During early food reintroduction, a caregiver also described academic workload and short school breaks as making scheduled enteral-nutrition intake difficult [(9), participant B2].

Illness visibility and peer interaction created additional pressure. Children reported concern about questions, unwanted attention, or being treated differently, and some managed disclosure by hiding or delaying intake. One child described drinking when classmates were not paying attention, hiding in the teachers’ office, or waiting until school ended [(19), participant N2]. Spiers (14) also documented embarrassment when an audible feeding-pump alarm made treatment visible in class. Adolescent-focused reports discussed autonomy, peer identity, and disclosure more explicitly than reports involving younger children; however, age-stratified conclusions were not possible because age-specific findings were not consistently reported.

### 3.7 Reported adaptation and changing perceptions during EEN

Direct child and adolescent accounts suggested that perceptions of EEN sometimes changed from early resistance or uncertainty towards partial acceptance, particularly when treatment benefits became noticeable. However, the contributing evidence was largely cross-sectional or retrospective, and the timing of data collection was inconsistently reported. These accounts should therefore be interpreted as reported changes in experience rather than as evidence of a universal, linear, or prospectively observed longitudinal trajectory.

Several reports suggested that treatment initiation could be especially difficult because children were simultaneously adjusting to formula intake, food restriction, symptoms, and disrupted routines. One adolescent stated, “This [EEN] is hard but it’s going to be worth it once I’m done” [(14), p. 59]. Because treatment timing was not consistently reported, the available evidence does not support the identification of a universal critical adaptation period.

Symptom relief, improved energy, perceived physical recovery, and recognition of treatment benefit appeared to support persistence for some children. One child reported that abdominal pain eased, stools became normal, weight increased, and running became possible [(18), participant N11]. An adolescent in Spiers (14) similarly described having more energy and feeling physically better. These accounts suggest that perceived treatment benefit may support persistence, but they do not establish causality or a standard adaptation timetable.

### 3.8 Support needs across EEN and early food reintroduction

Support findings came from all three perspective groups. Children emphasised emotional reassurance, practical encouragement, school understanding, and regular clinical contact. Caregivers described supervision and family-management work, while healthcare professionals contributed service-level concerns during early food reintroduction.

Children’s accounts and caregiver reports converged on the importance of companionship, reassurance, reminders, and practical help. One child described parental explanation and supervision as helping them continue EEN (18), while another advised involving family members or asking them to give up favourite foods during the EEN course (4). At the same time, adolescent and caregiver data indicated tension between supervision and developing autonomy. This finding should therefore be understood as a family-management issue rather than a recommendation for greater control.

Professional support included specific formula advice, symptom management, regular review, and guidance for early food reintroduction. Brown et al. reported the child statement “Being reviewed often by the medical team helps keep you on track.” Zhou et al. added multi-perspective evidence that food-reintroduction advice was often insufficiently specific. Because these strategies were described as needs rather than evaluated interventions, they are presented as priorities for service development and future testing.

## 4 Discussion

This review synthesised direct child/adolescent accounts together with clearly labelled caregiver and healthcare-professional perspectives. The three-domain structure—burden, reported adaptation and changing perceptions, and support—was stable in both the child-focused sensitivity analysis and the sensitivity analysis excluding the two survey-based reports, and ConQual confidence was moderate for all three findings.

Treatment burden theory, normalisation process theory, and the Family Management Framework were selected after completion of the inductive meta-aggregation as interpretive lenses for the Discussion. They did not guide eligibility, coding, category formation, or ConQual assessment. Each framework is used for a distinct purpose: treatment burden theory interprets the work imposed by EEN (20–22), normalisation process theory helps explain incorporation of a new regimen into daily life (23, 24), and the Family Management Framework illuminates family organisation and autonomy (25). These theoretical interpretations should not be attributed to the primary-study participants unless explicitly reported by those studies.

### 4.1 Multiple burdens of EEN

The multiple-burdens finding was supported mainly by direct child/adolescent accounts and had moderate ConQual confidence. Formula-related discomfort, interrupted eating, school constraints, and social visibility were recurrent across settings, although administration route and age-specific variation were insufficiently reported.

These findings suggest that apparent non-adherence should be distinguished from feasibility, acceptability, persistence, and treatment completion. From a *post hoc* treatment-burden perspective, incomplete intake may reflect a mismatch between treatment work and available physical, emotional, family, or environmental capacity rather than lack of motivation alone.

This interpretation is particularly important for paediatric clinical nutrition and nursing care. Eating is closely linked to family interaction, peer belonging, school participation, and social identity. When ordinary eating is interrupted, children may experience not only hunger or food cravings, but also a temporary loss of participation in normal childhood and adolescent life. Similarly, when formula drinking makes illness visible to classmates, children must manage both treatment requirements and concerns about being different. These findings show that the burden of EEN is simultaneously nutritional, bodily, emotional, practical, and social.

For clinical practice, teams may consider assessing sensory tolerance, gastrointestinal symptoms, emotional distress, food cravings, school constraints, and disclosure concerns in addition to prescribed formula volume.

### 4.2 Reported adaptation and changing perceptions during EEN

The finding concerning reported adaptation and changing perceptions had moderate ConQual confidence. Some children described or retrospectively recalled movement from early resistance towards greater acceptance as treatment benefits became noticeable. However, because the contributing studies were predominantly cross-sectional or retrospective and interview timing was inconsistent, this pattern should not be interpreted as a confirmed longitudinal trajectory, universal sequence, or stage model.

Normalisation process theory provides a useful *post hoc* lens for interpreting how children may understand and incorporate EEN into daily life. Early resistance may reflect the unfamiliarity and restrictiveness of treatment and its incompatibility with ordinary eating, school, and social routines. When symptom relief or physical improvement becomes noticeable, some children may increasingly recognise the value of the regimen, which may support motivation and persistence. Nevertheless, the synthesis does not establish a standard adaptation timetable or causal relationship between perceived benefit and adherence.

### 4.3 Support systems for sustaining EEN

The support-needs finding had moderate ConQual confidence and combined direct child data with family-management and service-level perspectives. Convergence across sources supports the relevance of emotional, practical, and informational support, while differences in perspective caution against treating caregiver or professional accounts as substitutes for children’s experience.

Parents and caregivers often performed reminders, preparation, transport, monitoring, and negotiation of family meals. The *post hoc* Family Management Framework helps interpret this work, including the need to balance safety and completion with adolescent autonomy. The synthesis does not support a single optimal level of supervision.

Professional support was also a major need identified in this review. Children and caregivers required practical, specific, and continuous guidance, particularly regarding formula administration, symptom management, school implementation, and food reintroduction. The food-reintroduction study also described uncertainty caused by a lack of standardised guidance. Contextual survey evidence outside the meta-aggregation similarly indicates that parent perspectives on EEN are shaped by clinician communication, cost, and practical feasibility (7).

Service teams may consider ensuring access to clear contact routes, practical formula advice, symptom-management support, school-related guidance, and specific food-reintroduction information.

### 4.4 Implications for clinical practices and service organisation

The synthesis supports three practices considerations: assessing EEN burden beyond prescribed formula volume; asking children directly about their emotional, social, and school-related experiences; and providing phase-specific practical guidance during active EEN and early food reintroduction. Professional education may also address developmental communication, family management, school participation, and the distinction among non-adherence, low acceptability, limited feasibility, interrupted persistence, and non-completion. Children’s direct accounts should be sought whenever developmentally appropriate.

At the service level, organisations may clarify responsibility for dietetic contact, symptom escalation, school communication, and food-reintroduction guidance. Potential responses include structured care pathways, monitoring tools, family coaching, and school liaison, tailored to the child’s developmental stage, treatment phase, and local context. School-related support should be individualised in collaboration with children, families, healthcare professionals, and educational staff.

These practices and service considerations are derived from the qualitative synthesis and should not be interpreted as established or validated interventions. Their feasibility, acceptability, equity, and clinical effectiveness require co-design and prospective evaluation.

### 4.5 Strengths and limitations

Strengths include explicit separation of direct, proxy, and professional perspectives; identification of treatment phase; an auditable 22-finding matrix with JBI credibility ratings; ConQual assessment; and two complementary sensitivity analyses. Inclusion of a thesis broadened coverage beyond journal articles, and item-level appraisal avoided a non-standard summary grade.

This review has several limitations. Only six reports were included, and methodological reporting—particularly reflexivity and philosophical positioning—was incomplete. Two reports contributed qualitative evidence from open-ended questionnaire responses rather than in-depth interviews; although the synthesis structure remained unchanged when these reports were excluded, their responses provided less contextual and interpretive depth and therefore warrant cautious use. English- and Chinese-language restriction may have excluded experiences from other food cultures and healthcare systems. Accordingly, themes concerning family meals, parental supervision and adolescent autonomy, treatment disclosure and peer interaction, school accommodation, dietetic access, and food-reintroduction practices may partly reflect context-specific cultural norms and service arrangements. Reports spanned 2005–2025, during which formulas, biologic pathways, dietetic access, family support, and school accommodations may have changed. Contextual details such as age-specific findings, disease activity, route, formula, completion, and interview timing were inconsistently reported, preventing route- or developmental-stage synthesis. Some categories drew on retrospective surveys or caregiver/professional perspectives. The review addressed active EEN, retrospective completion, and early food reintroduction, not long-term maintenance, relapse, repeated courses, or later dietary behaviour. A formal PRESS review was not undertaken. Although CINAHL was added during peer-review revision, APA PsycINFO could not be searched because it was unavailable through institutional access, so relevant psychology-indexed studies may have been missed. In addition, the July 2026 supplementary update did not constitute a complete rerun of all licensed databases, and records indexed only in those sources may therefore have been missed.

### 4.6 Priorities for future research

Longitudinal qualitative studies should follow children from treatment initiation through completion, food reintroduction, maintenance, relapse, and repeated EEN courses, with interview timing reported explicitly.

Future studies should prioritise children’s direct voices, use developmentally appropriate methods, and report age-stratified findings. Paired child-caregiver designs could examine agreement and divergence without treating proxy accounts as interchangeable.

Comparative work is needed across oral and nasogastric routes, formula types, cultural food practices, school systems, and models of dietetic or nursing support. Core contextual variables and treatment-completion outcomes should be reported consistently.

Candidate strategies arising from this synthesis, including structured initiation support, family coaching, school liaison, and food-reintroduction pathways, should be prioritised for co-design and prospective evaluation across diverse paediatric settings.

## 5 Conclusion

Across six reports, direct child and adolescent accounts supported a three-part synthesis of EEN burden, reported adaptation and changing perceptions, and support needs. Children described formula-related discomfort, interruption of normal eating, school constraints, and illness visibility. Some children reported greater acceptance when symptoms or energy improved, but the available evidence does not establish a universal, linear, or prospectively observed adaptation trajectory.

Caregiver and healthcare-professional perspectives added information about family work, service communication, and early food reintroduction. These perspectives are complementary and should not be presented as children’s lived experience. Supportive assessment, family partnership, school coordination, and practical dietary guidance emerged as practical priorities for development and evaluation.

ConQual confidence was moderate for all three synthesised findings. Conclusions remain constrained by the small, heterogeneous, geographically uneven, and temporally broad evidence base and should not be extrapolated to long-term maintenance, relapse, repeated EEN, or later nutritional behaviour.
