# Supplementary material for: Treatment burden, adaptation, and support needs during exclusive enteral nutrition in children with Crohn’s disease: a qualitative meta-synthesis
Source: Front Nutr. 2026 Sep 16;13:1920373. doi: 10.3389/fnut.2026.1920373 (PMC13625674; doi:10.3389/fnut.2026.1920373)
Supplement: Supplementary file 1 [file Supplementary_file_1.docx]

**Supplementary Material**

Treatment burden, adaptation, and support needs during exclusive enteral nutrition in children with Crohn’s disease: a qualitative meta-synthesis

**Manuscript ID: 1920373**

# **Supplementary Table S1. Complete database strategies and search update log**

| **Database / platform** | **Search date** | **Complete strategy / syntax** | **Limits** | **Records** |
| --- | --- | --- | --- | --- |
| PubMed / NCBI | 30 Jan 2026 | ((Crohn Disease[Mesh] OR Crohn*[tiab] OR inflammatory bowel disease*[tiab]) AND (Child[Mesh] OR Adolescent[Mesh] OR child*[tiab] OR adolescen*[tiab] OR pediatric*[tiab] OR paediatric*[tiab]) AND (exclusive enteral nutrition[tiab] OR enteral nutrition[tiab] OR liquid diet*[tiab] OR formula diet*[tiab] OR polymeric formula*[tiab] OR elemental formula*[tiab] OR oral feed*[tiab] OR nasogastric feed*[tiab] OR tube feed*[tiab]) AND (qualitative[tiab] OR interview*[tiab] OR experience*[tiab] OR perception*[tiab] OR acceptab*[tiab] OR feasib*[tiab] OR persist*[tiab] OR complet*[tiab] OR adapt*[tiab] OR support need*[tiab])) | English/Chinese; inception to date | 286 |
| Embase / Elsevier | 30 Jan 2026 | ('crohn disease'/exp OR crohn*:ti,ab OR 'inflammatory bowel disease*':ti,ab) AND ('child'/exp OR 'adolescent'/exp OR child*:ti,ab OR adolescen*:ti,ab OR pediatric*:ti,ab OR paediatric*:ti,ab) AND ('enteral nutrition'/exp OR 'exclusive enteral nutrition':ti,ab OR 'liquid diet*':ti,ab OR 'formula diet*':ti,ab OR 'polymeric formula*':ti,ab OR 'elemental formula*':ti,ab OR 'oral feed*':ti,ab OR 'nasogastric feed*':ti,ab OR 'tube feed*':ti,ab) AND ('qualitative research'/exp OR qualitative:ti,ab OR interview*:ti,ab OR experience*:ti,ab OR perception*:ti,ab OR acceptab*:ti,ab OR feasib*:ti,ab OR persist*:ti,ab OR complet*:ti,ab OR adapt*:ti,ab OR 'support need*':ti,ab) | English/Chinese; inception to date | 106 |
| Web of Science Core Collection / Clarivate | 30 Jan 2026 | TS=((Crohn* OR "inflammatory bowel disease*") AND (child* OR adolescen* OR pediatric* OR paediatric*) AND ("exclusive enteral nutrition" OR "enteral nutrition" OR "liquid diet*" OR "formula diet*" OR "polymeric formula*" OR "elemental formula*" OR "oral feed*" OR "nasogastric feed*" OR "tube feed*") AND (qualitative OR interview* OR experience* OR perception* OR acceptab* OR feasib* OR persist* OR complet* OR adapt* OR "support need*")) | English/Chinese; inception to date | 198 |
| Cochrane Library / Wiley | 30 Jan 2026 | (Crohn* OR "inflammatory bowel disease"):ti,ab,kw AND (child* OR adolescen* OR pediatric* OR paediatric*):ti,ab,kw AND ("exclusive enteral nutrition" OR "enteral nutrition" OR "liquid diet" OR "formula diet" OR polymeric OR elemental OR "oral feeding" OR nasogastric OR "tube feeding"):ti,ab,kw AND (qualitative OR interview OR experience OR perception OR acceptability OR feasibility OR adaptation OR support):ti,ab,kw | English/Chinese; inception to date | 2 |
| Scopus / Elsevier | 30 Jan 2026 | TITLE-ABS-KEY((Crohn* OR "inflammatory bowel disease*") AND (child* OR adolescen* OR pediatric* OR paediatric*) AND ("exclusive enteral nutrition" OR "enteral nutrition" OR "liquid diet*" OR "formula diet*" OR "polymeric formula*" OR "elemental formula*" OR "oral feed*" OR "nasogastric feed*" OR "tube feed*") AND (qualitative OR interview* OR experience* OR perception* OR acceptab* OR feasib* OR persist* OR complet* OR adapt* OR "support need*")) | English/Chinese; inception to date | 62 |
| CNKI | 30 Jan 2026 | 主题=(克罗恩病 OR 炎症性肠病) AND 主题=(儿童 OR 患儿 OR 青少年) AND 主题=(全肠内营养 OR 排他性肠内营养 OR 肠内营养 OR 液体饮食 OR 配方饮食 OR 多聚体配方 OR 要素配方 OR 经口喂养 OR 鼻胃管喂养 OR 管饲) AND 主题=(质性 OR 访谈 OR 体验 OR 感受 OR 认知 OR 适应 OR 可接受性 OR 可行性 OR 坚持 OR 完成 OR 支持需求) | Chinese/English; inception to date | 31 |
| Wanfang | 30 Jan 2026 | (克罗恩病 OR 炎症性肠病) AND (儿童 OR 患儿 OR 青少年) AND (全肠内营养 OR 排他性肠内营养 OR 肠内营养 OR 液体饮食 OR 配方饮食 OR 鼻胃管 OR 管饲) AND (质性 OR 访谈 OR 体验 OR 感受 OR 适应 OR 可接受性 OR 可行性 OR 依从 OR 支持需求) | Chinese/English; inception to date | 90 |
| VIP | 30 Jan 2026 | M=(克罗恩病 OR 炎症性肠病) AND M=(儿童 OR 患儿 OR 青少年) AND M=(全肠内营养 OR 排他性肠内营养 OR 肠内营养 OR 液体饮食 OR 配方饮食 OR 鼻胃管 OR 管饲) AND M=(质性 OR 访谈 OR 体验 OR 感受 OR 适应 OR 可接受性 OR 可行性 OR 依从 OR 需求) | Chinese/English; inception to date | 20 |
| SinoMed | 30 Jan 2026 | (克罗恩病 OR 炎症性肠病) AND (儿童 OR 患儿 OR 青少年) AND (全肠内营养 OR 排他性肠内营养 OR 肠内营养 OR 液体饮食 OR 配方饮食 OR 鼻胃管喂养 OR 管饲) AND (定性研究 OR 质性研究 OR 访谈 OR 体验 OR 感受 OR 适应 OR 可接受性 OR 可行性 OR 依从 OR 支持需求) | Chinese/English; inception to date | 67 |
| Structured supplementary update | 20 Jul 2026 | Original paediatric/EEN/qualitative concept blocks in PubMed; targeted searches of publisher, index, citation, and Chinese-language title sources for records published or indexed from 31 Jan to 20 Jul 2026 | English/Chinese; update period | 8 candidate records; 0 eligible |
| CINAHL with Full Text / EBSCOhost | 10 Aug 2026 | (Crohn* OR "Crohn disease" OR "Crohn's disease") AND ("exclusive enteral nutrition" OR EEN OR "enteral nutrition" OR "exclusive enteral feeding" OR "liquid diet" OR "formula diet" OR "polymeric formula" OR "elemental formula" OR "oral feeding" OR "nasogastric feeding" OR "tube feeding" OR "food reintroduction") AND (child* OR adolescen* OR pediatric* OR paediatric* OR teen* OR youth* OR "young people" OR parent* OR caregiver* OR famil* OR "healthcare professional*" OR "health care professional*" OR nurse* OR dietitian*) AND (qualitative OR interview* OR "focus group*" OR phenomenolog* OR "thematic analysis" OR "content analysis" OR "mixed method*" OR experience* OR perception* OR perspective* OR adapt* OR acceptab* OR feasib* OR adheren* OR persist* OR complet* OR "support need*" OR support* OR barrier* OR facilitator*) | English/Chinese; inception to date | 154 before deduplication |

## **Supplementary Table S1B. Execution record for the 2026 search update**

| **Source searched** | **Date** | **Search expression / procedure** | **Outcome** |
| --- | --- | --- | --- |
| PubMed / NCBI | 20 Jul 2026 | Original Crohn/paediatric/EEN/qualitative concept blocks AND (("2026/01/31"[Date - Publication] : "2026/07/20"[Date - Publication]) OR ("2026/01/31"[Entrez Date] : "2026/07/20"[Entrez Date])). | Records were screened against the full eligibility criteria and merged with targeted-search records. |
| Public publisher and index sites | 20 Jul 2026 | "exclusive enteral nutrition" AND (child* OR adolescen* OR pediatric* OR paediatric*) AND (qualitative OR interview* OR experience* OR perspective*) on official journal/publisher and indexing pages. | Potentially relevant 2026 EEN and paediatric IBD records were retrieved for full eligibility assessment. |
| Chinese-language title searches | 20 Jul 2026 | 克罗恩病 AND (儿童 OR 患儿 OR 青少年) AND (全肠内营养 OR 食物重新引入) AND (质性 OR 体验 OR 需求). | No additional eligible full-text qualitative report was identified. |
| Backward and forward citation checking | 20 Jul 2026 | Reference lists and citing records of the six included reports, relevant reviews, and the 2026 paediatric IBD qualitative meta-synthesis were checked. | Eight unique update candidates were screened after de-duplication; zero met all criteria. Reasons are reported in Supplementary Table S4. |
| Reference-list and thesis verification | 20 Jul 2026 | Reference lists of included reports and relevant reviews were checked. The Spiers thesis was verified in the Theses Canada catalogue and the complete 131-page full text was obtained. | The thesis met all substantive eligibility criteria and was appraised and included. |
| CINAHL with Full Text / EBSCOhost | 10 Aug 2026 | Full Boolean strategy reported in Supplementary Table S1; searched from inception to date. | 154 records retrieved; 122 duplicates removed against the existing library; 32 title/abstract records screened; 29 excluded; 3 reports assessed in full text; 3 excluded; 0 newly included. |
| APA PsycINFO | Peer-review revision | Considered as an additional psychosocial database. Institutional access provided APA PsycArticles and Psychology and Behavioral Sciences Collection, but not APA PsycINFO. | PsycINFO was not searched because it was unavailable through institutional access; the available databases were not treated as equivalent substitutes. This limitation is stated in the manuscript. |

The update was designed to reduce the interval between the original search and resubmission. It was not represented as a complete rerun of every licensed database interface, and this residual limitation is stated in the manuscript.

*Search-update note. The 20 July 2026 update was a structured supplemental search rather than a complete rerun of all licensed databases. PubMed/NCBI was the formal database component; targeted public publisher/index pages, Chinese-language title searching, and backward/forward citation checking were used as supplemental methods. Individual public publisher/index pages were not maintained as a separate export log and are therefore not presented as a complete database rerun.*

# **Supplementary Table S2. Detailed context of the six included reports**

| **Study / country** | **Qualitative contributors and perspective** | **Age / sex** | **Disease context and previous EEN** | **Route / formula / planned duration / completion** | **Setting and timing** | **Sampling, collection, and analysis** | **EEN-related evidence retained** |
| --- | --- | --- | --- | --- | --- | --- | --- |
| Wei et al. (2021), China | 12 children; direct child accounts | 7–15 y; 7 male, 5 female | Disease duration 0.25–60 months; 8 active, 4 remission; first induction EEN | Oral EEN; formula and planned duration NR; completion status NR | Home and school; interviewed during admission or follow-up after initial EEN | Purposive maximum-variation sampling; semi-structured interviews, 30–45 min; Colaizzi seven-step analysis | Taste/monotony, gastrointestinal symptoms, school barriers, peer stigma, parental supervision, self-regulation, and symptom improvement |
| Zhang et al. (2024), China | 10 children; direct child accounts | 8–14 y; 6 male, 4 female | 4 active, 6 remission; disease duration and previous EEN NR | Route/formula/duration NR; children with interrupted EEN excluded | Hospital interview room; during EEN pathway; exact timing NR | Purposive maximum-variation sampling; semi-structured interviews, 20–30 min; Colaizzi seven-step analysis | Desire for ordinary food, family-meal distress, emotional support, preparation/water/time barriers, and school concealment |
| Brown et al. (2023), New Zealand | 44 children; direct child responses | 5.4–16.5 y (mean 11.3); 24 male, 20 female | Mean 3.3 y since diagnosis; 10 had >1 EEN course; 81.8% remission at survey | Completed 8-week polymeric EEN; route NR; 59.1% receiving maintenance EN at survey | Routine outpatient review; interval from EEN completion varied | Convenience recruitment; 9-item survey with free-text questions; thematic analysis of verbatim responses | Flavour/taste/cravings, mental challenge, energy and symptom benefit, family, school, and clinical support |
| Svolos et al. (2017), United Kingdom | 29 children and 29 parents; child and caregiver perspectives; 51 open-ended comments | Median 13.3 y (IQR 11.1–15); 20 male, 9 female | Previous EEN during 2015; disease duration/activity NR | 8-week EEN; 55% used NG support; 79% completed; formula NR | Postal questionnaire after prior EEN; recall interval NR | Census of eligible families; separate child and parent questionnaires; open comments grouped by respondent and valence | Perceived difficulty, isolation, route-related acceptability, and willingness to repeat EEN |
| Spiers (2005), Canada | 10 adolescents; direct adolescent accounts | 13–16 y; 6 male, 4 female | All had Crohn’s disease and previous EEN; EEN received 1–4 times | Three on EEN and seven on supplemental EN at interview; NG and gastrostomy routes; formula NR; EEN described as 8–12 weeks | Home or IWK Health Centre; current and recalled treatment experiences | Purposive recruitment; individual in-depth interviews; member checking; thematic analysis; 327 quotations coded | Decision-making, food deprivation, tube/pump burden, school and peer visibility, coping, family support, and healthcare-team support |
| Zhou et al. (2025), China | 7 teenagers, 7 caregivers, and 11 professionals contributed to 24 interviews | Teenagers 13–17 y; sex of qualitative subset NR | All teenagers had EEN-induced remission before food reintroduction; CD duration 11–113 months | Prior EEN 1.5–9 months; route/formula NR; all continued PEN during reintroduction | Homes, offices, or community; 11 face-to-face and 13 video interviews, 28–81 min | Purposive and snowball sampling; semi-structured interviews; conventional content analysis | Guidance gaps, teenager food acceptance, caregiver control/autonomy, school limits, and PEN tapering practices |

# **Supplementary Table S3. Source-specific finding-to-illustration audit trail for the 22 findings contributing to synthesis**

| **ID** | **Study / page** | **Perspective / phase** | **Author-level finding** | **Supporting illustration (English translation; original Chinese where applicable)** | **Credibility** | **Credibility rationale** | **Category** | **Synthesised finding** |
| --- | --- | --- | --- | --- | --- | --- | --- | --- |
| F1 | Wei 2021, p.60 | Child / active EEN | Repeated intake produced taste fatigue and aversion. | “After drinking it a few times, I started to get tired of it; the smell made me want to vomit.” (N5) Original: “大概喝几次就开始腻了，闻到就想吐。” | Unequivocal | Direct child quotation explicitly describes repeated formula intake causing taste fatigue and nausea; no additional reviewer inference is required. | Sensory discomfort and physical intolerance | Multiple burdens of EEN |
| F2 | Wei 2021, p.60 | Child / active EEN | Gastrointestinal discomfort impeded formula intake. | “I wanted to drink it, but I could not. I felt very full, had stomach pain, kept vomiting, and had no appetite.” (N6) Original: “我也想喝，但是喝不下，很饱啊，肚子痛又一直吐，没胃口。” | Unequivocal | Direct child quotation explicitly reports fullness, abdominal pain, vomiting, and inability to continue intake, directly supporting gastrointestinal intolerance. | Sensory discomfort and physical intolerance | Multiple burdens of EEN |
| F3 | Svolos 2017, Results | Caregiver / recalled EEN | Administration route altered perceived difficulty. | “My child found the liquid diet easy as it was through the tube.” | Unequivocal | Direct caregiver comment explicitly links tube administration with perceived ease of the liquid diet, directly supporting route-related perceived difficulty. | Sensory discomfort and physical intolerance | Multiple burdens of EEN |
| F4 | Zhang 2024, p.2166 | Child / active EEN | Children strongly desired to return to ordinary eating. | “I could not stand it at first and really wanted to eat as I had before.” (N4) Original: “一开始受不了，好想像以前那样。” | Unequivocal | Direct child quotation explicitly states inability to tolerate the regimen initially and a desire to return to previous eating. | Deprivation from interrupted normal eating | Multiple burdens of EEN |
| F5 | Svolos 2017, Results | Caregiver / recalled EEN | Exclusion from ordinary eating could be socially isolating. | “The liquid-only diet was very isolating at times for my child.” | Unequivocal | Direct caregiver comment explicitly characterises the liquid-only diet as socially isolating for the child. | Deprivation from interrupted normal eating | Multiple burdens of EEN |
| F6 | Spiers 2005, p.64 | Adolescent / active EEN | Food cravings intensified the burden of not eating. | “I have to say dealing with the cravings of certain foods like pizza or stuff that I didn’t even like before I got cravings for.” (William) | Unequivocal | Direct adolescent quotation explicitly describes food cravings during EEN, directly supporting deprivation from interrupted eating. | Deprivation from interrupted normal eating | Multiple burdens of EEN |
| F7 | Wei 2021, p.61 | Child / active EEN | Short school breaks interfered with scheduled formula intake. | “I need to drink every two hours… there are only five minutes between classes… it is difficult to do.” (N4) Original: “喝奶要2个小时一次…每节课之间只有5 min…有点难做到。” | Unequivocal | Direct child quotation explicitly links short school breaks with difficulty meeting the prescribed two-hour formula schedule. | Environmental constraints | Multiple burdens of EEN |
| F8 | Zhang 2024, pp.2166–2167 | Child / active EEN | Access to hot water and distance within school created practical barriers. | “Our classroom is on the fourth floor, but water is only available on the first floor… it wastes time.” (N7) Original: “我们的教室在4楼，但是只有1楼可以打水…浪费时间。” | Unequivocal | Direct child quotation explicitly identifies distance to hot-water access at school as a time-consuming practical barrier. | Environmental constraints | Multiple burdens of EEN |
| F9 | Zhou 2025, p.7 | Caregiver / early food reintroduction | Academic workload and short breaks disrupted prescribed PEN schedules. | “It was unrealistic for her to drink nutrition solution every 2 h at school… so she ends up only drinking it twice a day.” (B2) | Unequivocal | Direct caregiver quotation explicitly links school workload and short breaks with reduced scheduled enteral-nutrition intake during reintroduction. | Environmental constraints | Multiple burdens of EEN |
| F10 | Wei 2021, p.61 | Child / active EEN | Peer ridicule made children reluctant to disclose formula use. | “I did not dare tell my friends I was drinking formula. They would laugh and ask why I still drank formula at my age.” (N8) Original: “在朋友之间不敢说我在喝奶粉，他们会笑我，说你怎么这么大了还喝奶粉啊？” | Unequivocal | Direct child quotation explicitly reports fear of peer ridicule about formula use, directly supporting illness visibility and social pressure. | Illness visibility and social pressure | Multiple burdens of EEN |
| F11 | Zhang 2024, p.2167 | Child / active EEN | Children concealed or delayed intake to avoid peer attention. | “I drink when classmates are not paying attention, hide in the teachers’ office, or wait until school ends.” (N2) Original: “我趁同学不注意的时候喝，或者躲在老师办公室喝，或者放学才喝。” | Unequivocal | Direct child quotation explicitly describes concealing or delaying formula intake to avoid classmates’ attention. | Illness visibility and social pressure | Multiple burdens of EEN |
| F12 | Spiers 2005, p.67 | Adolescent / active tube feeding | Audible feeding-pump alarms caused embarrassment at school. | “Sitting in class doing work you can always hear the sound of the pump going around and that is just annoying and also that’s embarrassing.” (Dorothy) | Unequivocal | Direct adolescent quotation explicitly identifies audible pump noise in class as embarrassing. | Illness visibility and social pressure | Multiple burdens of EEN |
| F13 | Brown 2023, p.168 | Child / recalled completed EEN | EEN was experienced as a substantial psychological challenge. | “It is more a mental game than a physical struggle.” | Unequivocal | Direct child free-text response explicitly frames EEN as primarily a mental rather than physical struggle. | Early resistance and psychological difficulty | Reported adaptation and changing perceptions during EEN |
| F14 | Spiers 2005, p.59 | Adolescent / active EEN | Children could view EEN as difficult while anticipating future benefit. | “This [EEN] is hard but it’s going to be worth it once I’m done and stuff.” (Michelle) | Unequivocal | Direct adolescent quotation simultaneously describes EEN as hard and anticipates future benefit, directly supporting difficult early experience with changing appraisal. | Early resistance and psychological difficulty | Reported adaptation and changing perceptions during EEN |
| F15 | Wei 2021, p.61 | Child / active EEN | Symptom and functional improvement reinforced persistence. | “My abdominal pain eased, my stool became normal, I gained weight, and I could run.” (N11) Original: “肚子痛缓解了，大便正常了…体重增了，可以跑步了。” | Unequivocal | Direct child quotation explicitly reports symptom and functional improvement, directly supporting perceived treatment benefit. | Perceived benefit and self-regulation | Reported adaptation and changing perceptions during EEN |
| F16 | Spiers 2005, p.60 | Adolescent / active enteral nutrition | Improved energy and well-being reinforced treatment value. | “I have had more energy and I have been feeling better about myself… physically and stuff like that. Better about myself.” (Pamela) | Unequivocal | Direct adolescent quotation explicitly reports increased energy and improved physical well-being, directly supporting perceived benefit. | Perceived benefit and self-regulation | Reported adaptation and changing perceptions during EEN |
| F17 | Wei 2021, p.61 | Child / active EEN | Parental explanations and supervision supported persistence. | “My mother told me formula could help my intestines recover; whether I wanted to or not, I had to continue.” (N4) Original: “我妈妈跟我说喝奶粉可以促进肠道恢复，不坚持也得坚持。” | Unequivocal | Direct child quotation explicitly attributes persistence to parental explanation and supervision. | Family emotional and practical support | Support needs across EEN and early food reintroduction |
| F18 | Brown 2023, p.168 | Child / recalled completed EEN | Family participation reduced isolation and supported treatment. | “Get your family to participate or to give up their favorite foods during your course of EEN.” | Unequivocal | Direct child free-text advice explicitly recommends family participation or shared food restriction, directly supporting practical/family support. | Family emotional and practical support | Support needs across EEN and early food reintroduction |
| F19 | Zhou 2025, p.6 | Teenager / early food reintroduction | Overly strict caregiver control provoked covert eating and resistance. | “They were being a bit too strict, so that made me want to sneakily eat some even more.” (A7) | Unequivocal | Direct teenager quotation explicitly links overly strict caregiver control with covert eating and resistance. | Family emotional and practical support | Support needs across EEN and early food reintroduction |
| F20 | Brown 2023, p.168 | Child / recalled completed EEN | Regular clinical review helped sustain engagement. | “Being reviewed often by the medical team helps keep you on track.” | Unequivocal | Direct child free-text response explicitly states that frequent medical-team review helps maintain treatment engagement. | Professional and school-linked guidance | Support needs across EEN and early food reintroduction |
| F21 | Zhou 2025, p.4 | Caregiver / early food reintroduction | Post-EEN advice was too general for practical use. | “There is no specific recipe, like a list of recommended foods.” (B1) | Unequivocal | Direct caregiver quotation explicitly states that post-EEN advice lacked a specific food list/recipe, directly supporting insufficient practical guidance. | Professional and school-linked guidance | Support needs across EEN and early food reintroduction |
| F22 | Zhou 2025, p.8 | Professional / early food reintroduction | Families tapered enteral nutrition without structured professional intervention. | “All patients were self-tapering at home after remission, and to be honest, we did not have much intervention.” (C11) | Unequivocal | Direct professional quotation explicitly states that patients self-tapered at home with little professional intervention, directly supporting a structured-guidance gap. | Professional and school-linked guidance | Support needs across EEN and early food reintroduction |

Note. JBI credibility was judged at the finding-illustration level. Unequivocal indicates that the illustration directly and unambiguously supports the author-level finding without additional reviewer inference; credible indicates reasonable support but some interpretation is required; unsupported indicates that the illustration is inadequate to support the finding. The 22 findings retained in the synthesis were judged unequivocal after source-level review. Original Chinese wording is retained alongside the verified English translation for Chinese-language source quotations where applicable.

# **Supplementary Table S4. Excluded, borderline, and update-search records**

| **Report** | **Reason considered** | **Final decision** |
| --- | --- | --- |
| Chuong et al. (2019) | Paediatric IBD dietary-practice interviews addressed food experience. | Excluded: participants were not undergoing prescribed EEN and EEN-specific findings could not be isolated. |
| Mehta et al. (2020) | Parent perspectives addressed communication and barriers to EEN. | Excluded from meta-aggregation: survey and chart comparison without an identifiable qualitative analytic process or finding-to-illustration pairs; retained as contextual evidence. |
| Burgess et al. (2020) | Paediatric dietitian questionnaire directly addressed EEN practice and food reintroduction. | Excluded from meta-aggregation: open/free-text items were present, but only descriptive and inferential statistical analyses were reported; no qualitative themes or finding-to-illustration pairs were provided. |
| Martin and O’Meara (2025) | Highly relevant patient-perspective EEN survey. | Excluded: conference abstract only, with insufficient full-text methodological and quote-level data for appraisal. |
| Nicholas et al. (2007) | Broader paediatric IBD lived-experience study included food restriction and tube-feeding comments. | Excluded: diagnosis-specific and EEN-specific sources of the relevant quotations could not be isolated. |
| Chuong et al. duplicate records | Duplicate full-text copies of the same 2019 report. | Excluded as duplicates. |
| Cao et al. (2022) | Qualitative study of nasogastric home enteral nutrition self-management. | Excluded: adult sample and long-term home EN rather than paediatric induction EEN. |
| Cai et al. (2018) | Rich qualitative data on self-administered nasogastric feeding. | Excluded: adult sample aged 26–58 years and long-term nasogastric EN. |
| Mutsekwa et al. (2021) | Qualitative EEN experiences, challenges, and enablers. | Excluded: adult participants only. |
| Wang et al. (2025) | Qualitative home EN self-management challenges. | Excluded: adult sample; EN could be exclusive or partial and concerned long-term home management. |
| deBruyn et al. (2026) | Child and parent qualitative study of general paediatric IBD management. | Excluded: no extractable EEN-specific experience. |
| Tischler et al. (2026) | Implementation of paediatric EEN. | Excluded: quantitative secondary registry analysis. |
| CINAHL supplementary search (10 Aug 2026) | 154 records were retrieved; 122 were duplicates, 32 unique records were screened, and 3 reports underwent full-text assessment. | No additional study was included. All 3 full-text reports failed the prespecified eligibility criteria; the aggregate screening outcome is reported without assigning unverified article-specific reasons. |

# **Supplementary Table S5. ENTREQ reporting checklist**

| **ENTREQ domain / item** | **Reported location** |
| --- | --- |
| Aim and review questions | Abstract; Introduction |
| Synthesis methodology and rationale | Section 2.1 |
| Search strategy, databases, dates, and limits | Section 2.2; Supplementary Table S1 |
| Study selection and eligibility | Sections 2.3–2.4; Figure 1 |
| Quality appraisal and reviewer process | Section 2.5; Table 1 |
| Data extraction, source perspective, and treatment phase | Section 2.7; Table 2; Supplementary Tables S2–S3 |
| Finding-to-illustration linkage and credibility | Section 2.7; Supplementary Table S3 |
| Inductive category development and synthesis | Section 2.7; Figure 2; Table 3 |
| Confidence assessment | Sections 2.7 and 3.5; Table 6 |
| Sensitivity analysis | Sections 2.7 and 3.5; Tables 4–5 |
| Study characteristics and transferability | Section 3.3; Table 2; Supplementary Table S2 |
| Participant quotations and source attribution | Sections 3.6–3.8; Table 3; Supplementary Table S3 |
| Limitations, reflexivity, and interpretive boundaries | Sections 4.1–4.6 |
| Practice implications distinguished from evaluated interventions | Section 4.4; Conclusion |

# Supplementary references

Chuong, K.H., Haw, J., Stintzi, A., Mack, D.R., O’Doherty, K.C., 2019. Dietary strategies and food practices of pediatric patients, and their parents, living with inflammatory bowel disease: a qualitative interview study. International Journal of Qualitative Studies on Health and Well-being 14, 1648945.

Mehta, P., Pan, Z., Furuta, G.T., Kim, D.Y., de Zoeten, E.F., 2020. Parent perspectives on exclusive enteral nutrition for the treatment of pediatric Crohn disease. Journal of Pediatric Gastroenterology and Nutrition 71, 744–748.

Burgess, D., Herbison, K., Fox, J., Collins, T., Landorf, E., Howley, P., 2020. Exclusive enteral nutrition in children and adolescents with Crohn disease: dietitian perspectives and practice. Journal of Paediatrics and Child Health. doi:10.1111/jpc.15220.

Martin, A., O’Meara, M., 2025. P0846 A patient’s perspective on the use of exclusive enteral nutrition for the treatment of paediatric Crohn’s disease. Journal of Crohn’s and Colitis 19(Suppl 1), i1615.

Nicholas, D.B., Otley, A., Smith, C., Avolio, J., Munk, M., Griffiths, A.M., 2007. Challenges and strategies of children and adolescents with inflammatory bowel disease: a qualitative examination. Health and Quality of Life Outcomes 5, 28.

Cao, P.J., Jia, J.W., Wang, Y., et al., 2022. Self-management experience of home enteral nutrition with nasogastric tube among patients with Crohn’s disease: a qualitative study. Military Nursing 39(7), 54–56, 65.

Cai, Q., Li, F., Zhou, Y., 2018. Experiences of Chinese patients with Crohn’s disease in the self-administration of nasogastric feeding: a descriptive qualitative study. PLoS ONE 13, e0201421.

Mutsekwa, R.N., Edwards, J.T., Angus, R.L., 2021. Exclusive enteral nutrition in the management of Crohn’s disease: a qualitative exploration of experiences, challenges and enablers in adult patients. Journal of Human Nutrition and Dietetics 34, 440–449.

Wang, J., Zhang, Z., Yang, M., 2025. Home-based enteral nutrition self-management challenges in young and middle-aged patients with Crohn’s disease: a qualitative study. BMJ Open 15, e096416.

deBruyn, J.C., Hart, L., MacKean, G., et al., 2026. Child and parent perspectives in IBD management: a literature review and qualitative study. Crohn’s & Colitis 360 8(1), otag020.

Tischler, L., Boerkoel, A., Cantez, S.M., et al., 2026. Implementation of exclusive enteral nutrition in pediatric Crohn’s disease: a secondary analysis of the CLARA registry study. Frontiers in Pediatrics 14, 1787736.
